# Quantitation by Portable Gas Chromatography: Mass Spectrometry of VOCs Associated with Vapor Intrusion

**DOI:** 10.1155/2010/278078

**Published:** 2010-09-22

**Authors:** Justin D. Fair, William F. Bailey, Robert A. Felty, Amy E. Gifford, Benjamin Shultes, Leslie H. Volles

**Affiliations:** ^1^Department of Chemistry, Indiana University of Pennsylvania, Indiana, PA 15701, USA; ^2^Department of Chemistry, University of Connecticut, Storrs, CT 06269, USA; ^3^INFICON, Inc., 2 Technology Place, East Syracuse, NY 13057, USA

## Abstract

Development of a robust reliable technique that permits for the rapid quantitation of volatile organic chemicals is an important first step to remediation associated with vapor intrusion. This paper describes the development of an analytical method that allows for the rapid and precise identification and quantitation of halogenated and nonhalogenated contaminants commonly found within the ppbv level at sites where vapor intrusion is a concern.

## 1. Introduction

Indoor air quality has become an ever increasing topic of interest in light of reports detailing building-related illnesses (“sick building syndrome”) that produce symptoms such as upper respiratory diseases, headaches, dizziness, and fatigue [1]. Indeed, the typical person spends approximately 90% of his day indoors [2]. A number of models have been proposed to address the processes in which indoor air contamination occurs via vapor intrusion [3–6]. In addition, environmental factors have been identified to include the following: proximity to source, presence of shallow ground water, soil type, fractured bedrock, chemical degradation (or oxidation), building construction style, as well as floor/utility line(s) condition [7]. Petroleum-based products and chlorinated hydrocarbons, as well as a variety of other volatile organic compounds (VOCs), can drift great distances above local water tables and enter dwellings via vapor intrusion from sources such as industrial sites or landfills [8–12]. 

Once an instance of vapor intrusion has been identified, monitoring the volatile organic chemicals contained in indoor air may be accomplished by following the compendium methods of the US EPA [13–15]; thus assisting to survey and mitigate the known hazard(s). These methods, which involve both passive and active sampling techniques, require time-consuming concentration periods and incorporate off-site laboratory analysis of multiple samples resulting in delayed overall analysis time [16]. The Massachusetts Department of Environmental Protection is one of a handful of US agencies to provide guidelines for long-term exposure to contaminated indoor air. Their defined limits, ranging from 0.001 to 187 ppbv per individual contaminate, provide the necessary guidance to incident commanders or super fund site coordinators to determine if a building is indeed fit to occupy once a site has been identified to suffer from sick building syndrome [17].

**Figure 1:**
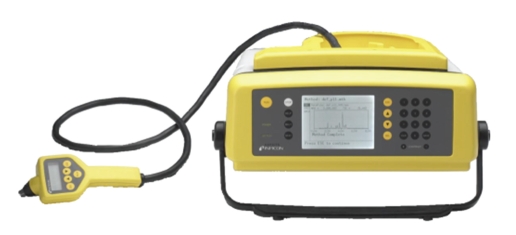
The HAPSITE Smart GC/MS system.

Field-portable instrumentation has been shown to offer rapid analysis of samples on site. Performing the chemical analysis onsite assists in eliminating sample integrity issues that may arise when volatile and hazardous samples are shipped long distances [18–20]. One field-portable gas chromatography/mass spectrometry (GC/MS) instrument in particular (Figure 1) utilizes a microconcentrator to sample ambient air at the ppbv level [21–24]. A previous modification to the instrument's existing sample flow path provided a means to identify multiple VOCs simultaneously with little to no analyte resolution in the total ion chromatogram (TIC). The entire sampling and analysis time takes less than 3 minutes [25]. This initial proof of concept demonstrated that shortened sample collection and sample run times did not hinder the portable mass spectrometer's identification of organic compounds. The present study was designed to evaluate the ability of this instrument to quantitate a mixture of VOCs commonly associated with vapor intrusion at the low ppbv level in accordance with current analytical methods [14]. 

## 2. Experimental Section

### 2.1. Reagents and Standards

Analytical grade standards in methanol solution (mix 1 and mix 2) were purchased from SPEX CertiPrep Inc. (Metuchen, NJ, USA). All standards purchased from SPEX CertiPrep Inc. arrived with a certificate of analysis detailing their final concentrations. The chemical standards used herein were chosen as they are “the primary sources of vapor intrusion problems in the United States” [7]. Nitrogen, UHP grade, was purchased from Airgas and used to dilute the standard mixes in Tedlar bags, purchased from SKC Inc. (Eighty Four, PA, USA). The samples used for construction of calibration curves were prepared by injecting 10.0 *μ*L of standard mix 1 (Table 1) into a Tedlar bag containing 1.00 L of UHP nitrogen. The samples used for the method detection limits (MDLs) determination were prepared by injecting 10.0 *μ*L of standard mix 2 (Table 2) into a Tedlar bag containing 1.00 L of UHP nitrogen. 

### 2.2. Portable GC Modifications

**Figure 2:**
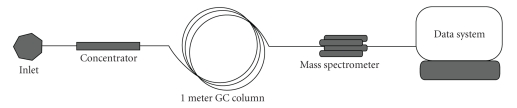
Schematic of the GC/MS with final modifications.

The gas chromatograph/mass spectrometer was modified as previously described [25]. However, instead of employing a Tenax microconcentrator as done previously, a TriBed microconcentrator, supplied by Inficon Inc. (East Syracuse, NY, USA), consisting of a thin-walled glass tube, packed with three layers of a proprietary blend of absorbents and wrapped with a heating element, was used to concentrate samples. This seemingly minor modification was essential for the trapping of the entire sample mixture, allowing complete sample adsorption for quantitation. The use of the multibed adsorbent, instead of a single absorbent, packed in order of increasing sorbent strength, provides adsorption of a wider range of compound classes as well as volatility ranges [26]. The sample flow path used in this study is depicted schematically in Figure 2. 

### 2.3. TriBed Microconcentrator Conditioning

The concentrator was cleaned using the following method: a 10 s line purge, a 3 s concentrator fill, a 15 s foreflush, an 11 s predesorb, a 30 s desorb, a 60 s foreflush, and a 124 s backflush (total 253 s run time). The concentrator was deemed to be “clean” when the TIC plot had a maximum value of ≤500,000 counts. If the maximum TIC count exceeded this value, the concentrator was repeatedly recleaned until this requirement was met. 

### 2.4. Portable GC Conditions

The following method was used for both blank runs and analyses of VOC samples: a 10 s line purge, a 15 s concentrator fill, a 1 s foreflush, a 20 s backflush, an 8 s pre-desorb, a 30 s desorb, a 43 s foreflush, and a 23 s backflush (total run time of 125 s). A mass range of 45 to 250 was selected, and the following temperatures were set: column, 65°C; GC/MS membrane, 65°C; valve oven, 65°C; probe, 40°C. A 15 s filament delay time was used to elute any ambient gasses such as oxygen, nitrogen, and carbon dioxide.

### 2.5. Linear Range and Method Detection Limit

The conditions outlined above were used in the creation of calibration curves for each analyte. A linear regression analysis of the average target-ion areas from four replicate runs at each concentration listed in Table 3 was used to construct a calibration curve for each of the analytes. For each sample, a concentrator clean, concentrator blank, and analytical run were performed sequentially. Calibration curves were constructed such that each curve was forced through the origin. 

Method detection limits were determined by diluting 10.0 *μ*l of each sample (Table 2) in 1.00 L of nitrogen. For each sample, a concentrator clean, concentrator blank, and analytical run were performed sequentially. Nine consecutive runs were used to evaluate each analyte's average concentration, variance, standard deviation, and method detection limit as described in the US Code of Federal Regulations (40CFR136, Appendix B). 

The calibration curve allowed quantitative library searching using the NIST mass spectral database. The following parameters were used in the NIST peak search: search window of 45 s, minimum reconstructed ion chromatogram (RIC) area of 28,000, minimum TIC area of 28,000, window expand factor of 0.05, minimum width of 7, minimum fit of 0.03, peak resolution of 5, maximum width of 70, minimum purity of 0.01, noise level mult of 2, and precedence level of 0. The following parameters were used in the automated mass spectral deconvolution and identification system search: analysis type simple, low mass of 45, high mass of 250, sensitivity of 30, resolution medium, and minimum match factor of 70. 

### 2.6. Audit Accuracy and Precision

Audit accuracy samples were prepared from individual standards in methanol solution supplied by SPEX CertiPrep Inc. (Metuchen, NJ, USA). A set of five randomly selected analytes, representing one-half of the total compounds of interest, were chosen to verify the accuracy of the calibration curve: xylenes, toluene, methylene chloride, tetrachloroethylene, and trichloroethylene. For each sample, a concentrator clean, concentrator blank, and analytical run were performed sequentially. Four consecutive runs were used to determine the accuracy and precision of the calibration curve. 

### 2.7. Breakthrough Times

**Figure 3:**
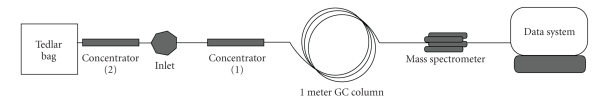
Configuration used for breakthrough study.

A clean TriBed microconcentrator was attached between the end of the instrument's probe and a Tedlar sample bag containing 10.0 *μ*L of sample mix 1 (Table 1) in 1.00 L of nitrogen. The configuration is illustrated in Figure 3. The Tedlar bag and concentrator were connected by a 0.4 cm section of Tygon tubing; sample flow rate was measured to be 99 mL/min. Each run consisted of a 10 s line purge and a 15 s concentrator fill to provide for a total concentration time for concentrator (2) of 25 s for each analytical run. As each successive run was performed, the bleed-through was caught by the main concentrator (1) and identified by the GC/MS system. 

## 3. Results and Discussion

### 3.1. Concentrator Selection

**Figure 4:**
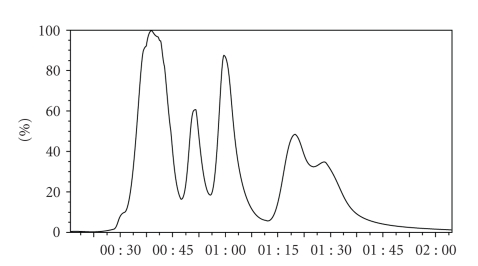
Total ion chromatogram (TIC) of analytes in mix 1 (Table 1) using a TriBed microconcentrator.

The TriBed microconcentrator was chosen for its ability to trap low molecular weight and low boiling analytes. The TriBed microconcentrator employs a bed consisting of multiple adsorbents of varying polarity to trap a wide range of analytes. The total ion chromatogram obtained from the 13 analytes in mix 1 (Table 1) is displayed in Figure 4. It might be noted that there is a small shoulder at ~30 s corresponding to vinyl chloride and ethyl chloride. In our experience, these two volatile analytes were difficult to trap using unisorbant concentrators such as the Tenax microconcentrator. The use of the multibed concentrator was essential in allowing the quantitation of all analytes in this study. 

### 3.2. Method Validation

**Figure 5:**
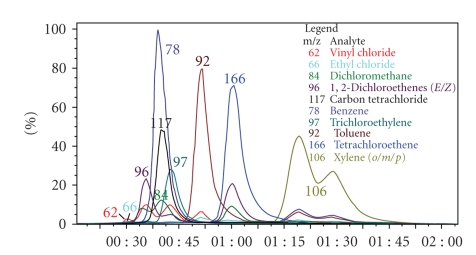
Reconstructed ion chromatogram (RIC) from the TIC illustrated in Figure 4.

A series of several calibration concentrations from 49.91 to 501.5 ppbv (Table 3) were sampled in four replicate runs at each concentration. A representative TIC and an RIC from a typical analysis of the 13 analytes in mix 1 (Table 1) are illustrated in Figures 4 and 5, respectively. The target ion, retention time, correlation coefficient, percent relative standard deviation, linear range, average response factor, and method detection limit for each analyte are summarized in Table 4.

It should be noted that all correlation coefficients are greater than 0.985. Moreover, the linear range for all but three of the analytes (*viz*, ethyl chloride, methylene chloride, and vinyl chloride) was 0–500 ppbv. The percent relative standard deviation (% RSD) ranged from 0.76% to 17.30%. The low-average response factor observed for ethyl chloride is a consequence of using a mass (m/z) of 66 rather than m/z = 64 for quantitation of this analyte; m/z = 66 was selected to provide clean calibration profiles while avoiding interference from other analytes in this study. 

The method detection limit (MDL) for each analyte was evaluated and they are summarized in Table 4 in units of ppbv and mg/m^3^. The MDL can also be viewed as the mass of each analyte in the total sample and these are also summarized in Table 4 for each analyte. The goal of this project was to have a total sampling and analysis time of less than 3 min; however, it should be noted that the MDL may be lowered with longer concentration times since the MDL is proportional to the sampling time. Thus, a majority of the MDL values can be brought below a 0.5 ppbv level using concentration times ranging from 30 s to 90 s. 

### 3.3. Determination of Audit Accuracy

The precision and accuracy of the newly determined analytical method were determined using randomly chosen analytes. The results of this method validation can be compared to the US EPA's Compendium Method TO-15 [14] that contains current indoor air monitoring guidelines as well as performance criterion for replicate precision and audit accuracy. Thus, the replicate precision was found by calculating the absolute difference between replicate measurements. Current indoor air methods are required to have a replicate precision with a percent difference less than or equal to 25% [14]. The audit accuracy, the degree of agreement with audit samples, was calculated by finding the percent difference between the nominal concentration and that of the audit sample. Current indoor air methods are required to have an audit accuracy less than or equal to 30% [14]. The concentration averaged over 4 replicate runs, along with the percent difference for the replicate precision and audit accuracy, are summarized in Table 5. The replicate precision ranged from 2% to 5% and the percent audit accuracy ranged from 3% to 26%. Both the replicate precision and the audit accuracy are within current US EPA method guidelines for the monitoring of indoor air.

### 3.4. Breakthrough Study

Using the instrument configuration depicted in Figure 3 and a sequence of analytical runs consisting of 10 s line purge and 15 s concentrator fill, breakthrough times for each analyte were determined. Not unexpectedly, the results summarized in Table 6 demonstrate that the most volatile compounds have the shortest breakthrough times and the need for a multibed microconcentrator.

## 4. Conclusion

A portable robust GC/MS fitted with a TriBed microconcentrator has been developed for identification and accurate quantitation of volatile organic compounds at the ppbv level. The method described herein adheres to the performance criteria described by the US EPA for the monitoring of indoor air. The collection and analysis of samples are accomplished in 3 min; concentrations of sub-ppbv analytes may be obtained with longer sampling times. It will be necessary to demonstrate that the instrument performs as expected in on-site monitoring of indoor air samples, but the present results suggest that the present analytical method should prove to be a powerful tool for the environmental monitoring of indoor air. 

## Figures and Tables

**Table 1:** Composition of standard mix 1 used for construction of calibration curves.

Compound	Concentration in CH_3_OH (*μ*g/mL)
Benzene	160
Carbon tetrachloride	314
Ethyl chloride	132
*Z*-1,2-Dichloroethene	99.0
*E*-1,2-Dichloroethene	99.0
Methylene chloride	174
Trichloroethylene	273
Vinyl chloride	128
Tetrachloroethylene	340
Toluene	189
*m*-Xylene	72.7
*p*-Xylene	72.7
*o*-Xylene	72.7

**Table 2:** Composition of standard mix 2 used for method detection limits determination.

Compound	Concentration in CH_3_OH (*μ*g/mL)	Concentration in *N*_2_ (ppbv)
Benzene	1.8	5.6
Carbon tetrachloride	3.5	5.6
Ethyl chloride	23.1	87.7
*(E*/*Z*)-1,2-Dichloroethene	4.4 (2.2 of each isomer)	11
Methylene chloride	1.9	5.5
Trichloroethylene	3.0	5.5
Vinyl chloride	22.4	87.6
Tetrachloroethylene	3.8	5.6
Toluene	2.1	5.6
*o,m, *and* p*-Xylene	2.4 (0.8 of each isomer)	5.5

**Table 3:** Concentrations used to construct calibration curves.

Compound	Concentration in *N*_2_ (ppbv)				
Benzene	50.08	125.2	250.4	375.6	500.8
Carbon tetrachloride	49.91	124.8	249.6	374.3	499.1
Ethyl chloride	50.03	125.1	250.2	375.2	500.3
(*E*/*Z*)-1,2-Dichloroethene	49.94	124.9	249.7	374.5	499.4
Methylene chloride	50.09	125.2	250.5	375.7	500.9
Trichloroethylene	50.04	125.1	250.2	375.3	500.4
Vinyl chloride	50.07	125.2	250.4	375.6	500.8
Tetrachloroethylene	50.13	125.3	250.7	376.0	501.3
Toluene	50.12	125.4	250.8	376.1	501.5
Xylene (*o*/*m*/*p*)	49.97	124.9	249.9	374.8	499.7

**Table 4:** Method validation results.

Compound	Mass (m/z)	*t*_*R*_^a^ (s)	*R* ^2^	% RSD	Linear Range (ppbv)	Avg. Response Factor	MDL		
							ppbv	mg m^−3^	ng sample^−1b^
Benzene	78	41	0.999	5.9	0–501	9.03 × 10^4^	3.2	10	0.25
Carbon tetrachloride	117	42	0.999	2.5	0–499	5.11 × 10^4^	2.7	11	0.28
(*E/Z*)-1,2-Dichloroethylene	96	36	0.987	11.3	0–499	2.40 × 10^4^	12.1	47.9	1.19
Ethyl chloride	66	34	0.990	17.3	0–250	4.14 × 10^3^	16.5	43.6	1.08
Methylene chloride	84	35	0.985	14.1	0–251	1.53 × 10^4^	27.6	96.0	2.38
Trichloroethylene	97	45	0.998	6.8	0–500	2.85 × 10^4^	2.1	11	0.27
Tetrachloroethylene	166	63	0.999	0.8	0–501	9.81 × 10^4^	0.7	4	0.1
Toluene	92	54	0.998	6.8	0–502	7.78 × 10^4^	1.8	6.9	0.17
Vinyl chloride	62	34	0.999	3.3	0–250	1.50 × 10^4^	13	33	0.81
*o, m, p*-Xylenes	106	82^c^	0.999	5.3	0–500	6.58 × 10^4^	3.2	13	0.34

^a^Retention time. ^b^Sample concentration time of 15 s at a 99 cm^3^ min^−1^ flow rate. ^c^A *t*_*R*_ of 82 s with a window wide enough to include all isomeric xylenes.

**Table 5:** Audit accuracy.

Compound	Concentration of standard (ppbv)	Calculated concentration (ppbv)	Replicate precision (% difference)	Percent audit accuracy (% difference)
Methylene chloride	173	128	2.52	25.9
Trichloroethylene	45.8	40.6	4.04	11.3
Tetrachloroethylene	16.2	13.1	3.16	19.6
Toluene	74.3	88.3	2.09	18.8
*o, m, p*-Xylenes	89.4	91.3	4.71	2.91

**Table 6:** Breakthrough study results.

Compound	Mass (m/z)	Breakthrough time (s)
Benzene	78	75
Carbon tetrachloride	117	350
(*E*/*Z*)-1,2-Dichloroethylenes	96	75
Ethyl chloride	66	25
Methylene chloride	84	50
Trichloroethylene	97	125
Tetrachloroethylene	166	>575
Toluene	92	150
Vinyl chloride	62	50
*o, m, p*-Xylenes	106	>575
